# Seasonal Changes in Free Amino Acid and Fatty Acid Compositions of Sardines, *Sardina pilchardus* (Walbaum, 1792): Implications for Nutrition

**DOI:** 10.3390/foods9070867

**Published:** 2020-07-02

**Authors:** Vida Šimat, Imen Hamed, Sandra Petričević, Tanja Bogdanović

**Affiliations:** 1 Department of Marine Studies, University of Split, Ruđera Boškovića 37, HR-21000 Split, Croatia; imen_hh@yahoo.fr; 2 Regional Veterinary Institute Split, Croatian Veterinary Institute, Poljička cesta 33, 21000 Split, Croatia; petricevic.vzs@veinst.hr (S.P.); t.bogdanovic.vzs@veinst.hr (T.B.)

**Keywords:** *Sardina pilchardus*, essential amino acids, omega-3, polyunsaturated fatty acids, proximate composition, Mediterranean diet

## Abstract

The aim of this study was to clarify the seasonal variation in the proximate composition of the free amino acid (AA) and fatty acid (FA) profiles of the European sardine (*Sardina pilchardus*) from the Adriatic Sea and to better understand the nutritive value needed to organize more effective industrial processing, aquaculture use and to ensure the health benefits for consumers through available bioactive compounds such as omega-3 FA and essential AA. The lipid content ranged from 1.18 to 10.58% during the year, being the highest from July to September. For the first time, this paper reports the monthly variation in AA content in sardines. The highest total AA content was measured during the winter period, from January (843 mg/100 g fillet) to March (953 mg/100 g) with histidine, arginine and threonine being the most dominant. The total content of essential free AA (histidine, threonine, valine, methionine, isoleucine, leucine, phenylalanine, tryptophan and lysine) ranged from 137 to 571 mg/100 g fillet (wet weight), recorded in May and March, respectively. The fatty acid profile analyses revealed the major saturated FA as palmitic (C16:0), followed by myristic (C14:0), and stearic (C18:0) acids, and the predominant monosaturated FA as oleic (C18:1*n–9*) and palmitoleic (C16:1*n–7*). The high concentrations of polyunsaturated FA in sardines were omega-3 FA, particularly eicosapentaenoic (EPA; 20:5*n-3*) and docosahexaenoic (DHA; 20:6*n-3*) FA. From July to September, their content was the highest (>3.5 g/100 g of sardine fillets), confirming that these species are excellent sources of bioactive lipids.

## 1. Introduction

The European sardine (*Sardina pilchardus*, Walbaum 1792) is a highly nutritive and the most important species of the Mediterranean Sea fisheries [1]. It is a small, oily, pelagic fish from the Clupeidae family distributed throughout the North Mediterranean, Adriatic, and Black Seas [2,3]. Sardines are caught all year around, with the highest peaks in the autumn–winter period. Both sardines and their byproducts have the potential to be used as nutraceuticals and pharmaceuticals and, for this reason, they are gaining more attention [1,4,5]. The nutritional importance of sardines as essential lipids rich in long-chain polyunsaturated fatty acids (PUFA), high-value, easy digestible proteins which contain all essential amino acids (AA) necessary for healthy human diets, minerals and vitamins, are well known [6,7]. These features give the fish health-promoting properties, including physical and mental wellbeing. For instance, the benefits from fish intake include a reduction in coronary heart disease risk and strokes in adults, the proper fetal development, and the improvement in cognitive development in infants and young children. The essential AA in marine proteins are highly exploitable as the digestibility of most seafood proteins exceeds 90% [8]. In general, proteins from fish have a noticeably high amount of lysine and leucine. Of the nonessential AA, glutamic acid, aspartic acid, and alanine are usually present in very high concentrations in marine protein sources [9]. The consumption of fish contributes to a reduction in protein–energy malnutrition, which is responsible for negative effects on respiratory and brain functions, the immune system, and which even causes death in both children and adults each year [10]. The nutritional benefits from fish consumption are also related to polyunsaturated fatty acids (PUFA), in particular, omega-3 eicosapentaenoic (EPA; 20:5*n-3*) and docosahexaenoic (DHA; 20:6*n-3*) fatty acids (FA), which are essential for human development and play an important role in the prevention and decreasing of many health disorders. EPA and DHA are essential for the growth, development, and improvement of human health [8]. Other positive effects have been reported, including the prevention of Alzheimer’s disease, dementia, osteoporosis, and obesity [11].

The proximate composition and nutritive value of small pelagic fish such as sardine, herring or anchovy vary greatly with the fishing season [12,13,14,15,16,17,18,19]. The intrinsic (species, size, and sexual maturity) and extrinsic parameters (food resource, water salinity, and temperature) particularly influence the composition of amino acids and fatty acids, affecting the nutritive value of the fish [20,21]. The lipid content is usually lower in periods of high energy demand (spawning or migration) in which the lipid reserves are used, and this affects the fatty acid profile [3], while seasonal changes in free amino acids for European sardines have not been reported. Given the importance of sardines to both consumers and the industry, there is a need for information on the changes in free amino acid and fatty acid compositions during the fishing season. The seasonal variation in the proximate composition and fatty acid profile of different sardine species has been reported [19,22,23,24,25,26]; however, to the best of our knowledge, there are no published reports on the seasonal changes in free amino acid composition of European sardines. Therefore, the aim of this study is to investigate the seasonal changes in proximate composition, free amino acid, and fatty acid profiles in the European sardine over a one-year period and to provide a report that is useful for dietary guidance and nutrition counseling.

## 2. Materials and Methods 

Approximately 8 kg of sardines were purchased from the local fish factory, which cooperates with large number of fishermen, between 15th and 20th of each month from January to December of 2018. The fish were caught in the central Adriatic fishing region (FAO 37.2.1) southwest of the long island by six to eight different fishing vessels. At every sampling point, sardines were placed in self-draining polystyrene boxes, packed in flake ice and delivered to the laboratory on the same day. To eliminate the effect of the fish size on the results, individual fish were selected to satisfy the criteria of 38–40 fish per kilo. The fish were gutted, filleted, and the fillets grinded using a laboratory blender (KINEMATICA Mikrotron MB 550, Switzerland). The grounded mass was used as the representative samples for the analyses, and the results were expressed as particular content/edible portion (EP). 

### 2.1. Proximate Composition of Fish 

The proximate composition was determined as the water content (drying the samples to constant weight at 105 °C), crude protein (Kjeldhal method, N × 6.25), and crude ash (calcination at temperatures ≤500 °C) [27]. The total lipid content was determined using the method of Bligh and Dyer [28]. The results are given as the mean of six repetitions ± standard deviation and expressed as three significant figures.

### 2.2. The AA Profile 

The free AA profile was determined by reverse-phase HPLC using previous derivatization with phenylisothiocianate (PITC) [29]. Briefly, 5 g of ground sardine fillet was homogenized with 20 mL of 0.01 N HCl using a mini-rotator Bio RS-24 (bioSan, Riga, Latvia) for 8 min at 4 °C and centrifuged at 10,000 g for 20 min. The supernatant was filtered through a regenerated cellulose (RC) filter and collected for further processing. In total, 250 μL of extract plus 50 μL of nor-leucine (1 mM used as internal standard) were deproteinized by adding 750 μL of acetonitrile, left to stand for 30 min and centrifuged at 10,000 g for 3 min. The free amino acids of the supernatant were derivatized [30] and analyzed using a 1290 Series Agilent Chromatograph (Agilent Technology, Palo Alto, CA, USA) with a photodiode array detector (254 nm). The phenyl isothiocarbamyl derivatives of amino acids were separated using a Novapack C18 (3.9 × 300 mm, 4 µm) column (Waters, Milford, MA, USA) at 52 °C. The solvent system consisted of solvent A—0.07 M sodium acetate adjusted to pH 6.55 with glacial acetic acid and containing 2.5% acetonitrile and solvent B—acetonitrile: water: methanol (45:40:15). The flow rate was 1 mL/min and the solvent gradient was as described by Flores et al. [31]. The results are given as the mean of four repetitions ± standard deviation and expressed in mg/100 g of EP, as three significant figures.

### 2.3. The FA Composition 

The relative fatty acid composition of sardine fillets was analyzed from Bligh and Dyer lipid extracts [4,5]. Briefly, the fatty acid methyl esters (FAME) were prepared by transmethylation using 2M KOH in methanol and heptane and measured using a gas chromatograph (model 3900; Varian Inc., Lake Forest, CA, USA) equipped with a flame ionization detector and a capillary column, 100 m × 0.25 mm; 0.2 μm film thickness (Restek). A Supelco mix set of 37 components (Sigma-Aldrich) was used as a FAME standard. The FA analyses were done in quadruplicate. The results obtained as percentages of methyl esters of certain fatty acids, calculated as the ratio of the peak area of interest and the total area of all peaks, were converted to g/100 g EP using corresponding Sheppard factors, as described in Food and Agriculture Organization/International Network of Food Data System (FAO/INFOODS) guidelines [32]. The results are expressed as three significant figures.

## 3. Results and Discussion

### 3.1. Proximate Composition of Sardine

The results of the seasonal changes in four major constituents of the EP of sardines are shown in Table 1. The crude protein (19.0–20.2%) and ash (1.45–2.30%) content remained relatively stable over the whole year. However, a significant seasonal variation was noticed concerning the moisture (69.4–78.8%) and crude lipid content (1.18–10.6%). Low lipid contents (<2%) were observed from January to March. As for most clupeids, the spawning season of European sardines from the Central Adriatic occurs between November and April, with a peak in winter, particularly in January and February, depending on a series of environmental factors such as temperature and salinity [33,34]. The low lipid content could be explained by a high energy demand during the spawning period [35,36]. The highest values of lipid content (>10%) were observed in the summer, which coincides with the feeding period of sardines, as there is a peak in primary production (planktonic blooms) during spring and summer. Sardines have an omnivorous diet that consists of zooplankton (decapods, crustacean and fish eggs, copepods, and cirripedes) and phytoplankton (dinoflagellates and diatoms). While zooplankton is used by sardines as a source of protein, microalgae are considered responsible for their lipidic fraction [37]. The inverse proportionality of the lipid and moisture content has also been reported for European anchovies (*Engraulis encrasicolus*) from the Central Adriatic [15].

Previously reported seasonal studies on *S. pilchardus,* particularly from the Mediterranean Sea and from Téboulba and Kélibia ports in Tunisia, found higher contents of lipids in spring–summer with a maximum of 7.8% and a minimum of 1.5% in the winter [26]. According to research conducted in Greece, in which *S. pilchardus* samples were taken from a local fish market in Thessaloniki, protein contents were reported as stable during seasons, within the range of 17.3–18.2% throughout the year [25]. Bouderoua et al. [24] investigated seasonal variations in *S. pilchardus* from the west Algerian coast, and found that dry matter, protein, and lipid contents were higher in summer with the lipid fraction being five times higher in comparison to winter. The lipid content in June was 10.5–11.5% depending on the fishing area. The variation in protein amounts was also higher in summer than in winter, ranging between 14.9% and 19.4%. We found no significant variation in protein contents related to the seasons. Significant changes were noticed in the lipid contents of *S. pilchardus*, caught off the Portuguese coast, with a minimum in March 1.2% and a maximum of 18.4% in September–October [38].

### 3.2. Free AA Composition of Sardines

Fish are a complete dietary source of 20 AA that serve as building blocks of protein and all 20 protein AA and their metabolites are required for normal cell physiology and function [39]. In terms of significance for humans, AA are traditionally grouped as nutritionally essential (histidine, arginine, isoleucine, leucine, lysine, methionine, phenylalanine, threonine, tryptophan, and valine), nonessential (asparagine, alanine, aspartic acid, tyrosine, and serine) or conditionally essential (taurine, glutamine, glutamic acid, glycine, and proline) AA [40]. Arginine is considered an essential AA for vascular homeostasis, spermatogenesis, and fetal growth of young mammals, as its dietary deficiency can result in metabolic, neurological or reproductive dysfunction [39]. The functional AA are those that regulate key metabolic pathways and are associated with the prevention and treatment of metabolic diseases (obesity and diabetes), infectious diseases, infertility, growth limitation, and organ dysfunctions (intestinal, neurological and renal) and, in human nutrition, these include arginine, cysteine, glutamine, leucine, proline, and tryptophan [39]. The seasonal changes in AA in sardines have not been reported thus far.

The changes in free AA profile in the EP of European sardines over a one-year period are shown in Table 2. Among the analyzed AA in sardines, only serine was not detected and among other AA, the seasonal variation was significant. 

**Figure 1:**
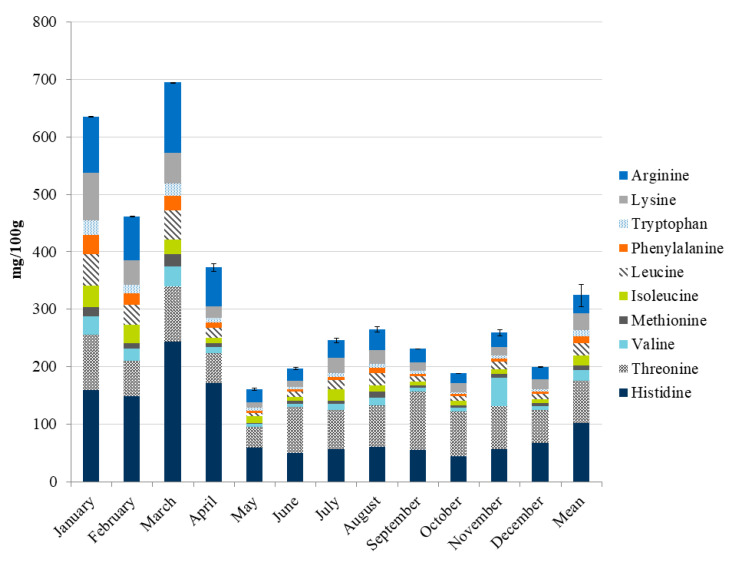
The sum of essential free AA, mg/100 g of arginine, histidine, isoleucine, leucine, lysine, methionine, phenylalanine, threonine, tryptophan, and valine) in sardine fillets over a one-year period.

The highest total AA content was measured during the winter period, from January to March. In this period histidine, arginine and threonine were the dominant essential AA in sardine EP. Lysine was also found in high concentrations in January. During spring and summer, the content of free AA was much lower, but histidine, threonine and arginine remained dominant. The seasonal changes in the total content of essential AA (histidine, threonine, valine, methionine, isoleucine, leucine, phenylalanine, tryptophan and lysine) are shown in Figure 1.

From January to May, and in December, the histidine levels contributed the most to the total essential AA content, from 18% to 32%, while from June to November, threonine’s contribution is dominant, from 19% in August to 36% of the total essential AA in September. The high levels of histidine are common in the muscle tissue of the migratory pelagic fish such as tuna, anchovy, sardine, mackerel, and herring, since it could have a buffer effect, protecting the tissues from the sudden increase in lactic acid produced during bouts of anaerobically powered muscle activity [41]. Aside from its multiple roles in protein interaction, histidine has many systemic and anti-inflammatory functions in the body. It is needed for the growth and repair of tissue, for the maintenance of myelin sheaths, for the improvement of the blood flow, as well as for removing surplus heavy metals from the body and for protection from radiation. It aids memory and cognitive function, as well as the immune system, and it is crucial in modulating inflammatory response as well as gastric acid regulation. Histidine is a precursor to histamine and several hormones (e.g., thyrotropin-releasing hormone) and critical metabolites affecting renal function and neurotransmission [40]. It performs important anti-inflammatory, antioxidant, and anti-secretory functions within the body and its deficiencies are related to anemia, chronic kidney disease, and allergic reactions [42,43]. Arginine was found in high content in the sardines (Table 2), particularly from January to March. As an essential AA, arginine plays an important role in the human body. It is involved in cell division, wound healing, ammonia removal, immune function, and hormone release processes. It also stimulates the biological synthesis of nitric oxide, a short-lived compound involved in circulatory and immune function, which plays important roles in neurotransmission, vasodilation, blood clotting, and the maintenance of blood pressure. For this reason, it is beneficial during recovery from a number of diseases such as sepsis, preeclampsia, hypertension, erectile dysfunction, anxiety, and it promotes vascular health [40].

In the winter period, sardines are a very rich source of essential and functional AA (Table 2, Figure 1). Threonine participates in the maintenance of strong bones and teeth. It accelerates wound healing and reduces fat in the liver. Threonine was also implicated in the support of various functions such as cardiovascular functions, the central nervous and immune systems, and the growth of the thymus. Methionine, on the other hand, is used as a treatment in liver disorders, depression, allergies, asthma, copper poisoning, and drug withdrawal. The primary function of isoleucine in the body is to boost the energy levels and to help the body recover after intense physical activity; thus, it is recommended to athletes. In addition, it is important in hemoglobin synthesis. Leucine is an energy supplier; it helps blood sugar regulation and muscle recovery after exercise. It is used clinically to improve body healing and it also affects brain functions. Lysine is used in drug preparations for the prevention and treatment of cold sores. Valine is associated with tissue repair and muscle coordination. Tryptophan is an important natural mood-lifting substance. It also helps with weight loss and overcoming sleep disorders. Phenylalanine plays a role in mood regulation and it promotes balanced neural and cognitive functions. Tyrosine supplements can help suppress appetite, support weight loss, and improve memory. They are also used for the treatment of a genetic disorder known as phenylketonuria [8]. According to FAO [44], the required dietary amounts of the nine essential AA change throughout life, and because cysteine can replace approximately 30% of the requirement for methionine, and tyrosine about 50% of the requirement for phenylalanine, these AA must also be considered. 

### 3.3. Fatty Acids Composition of Sardines

**Figure 2:**
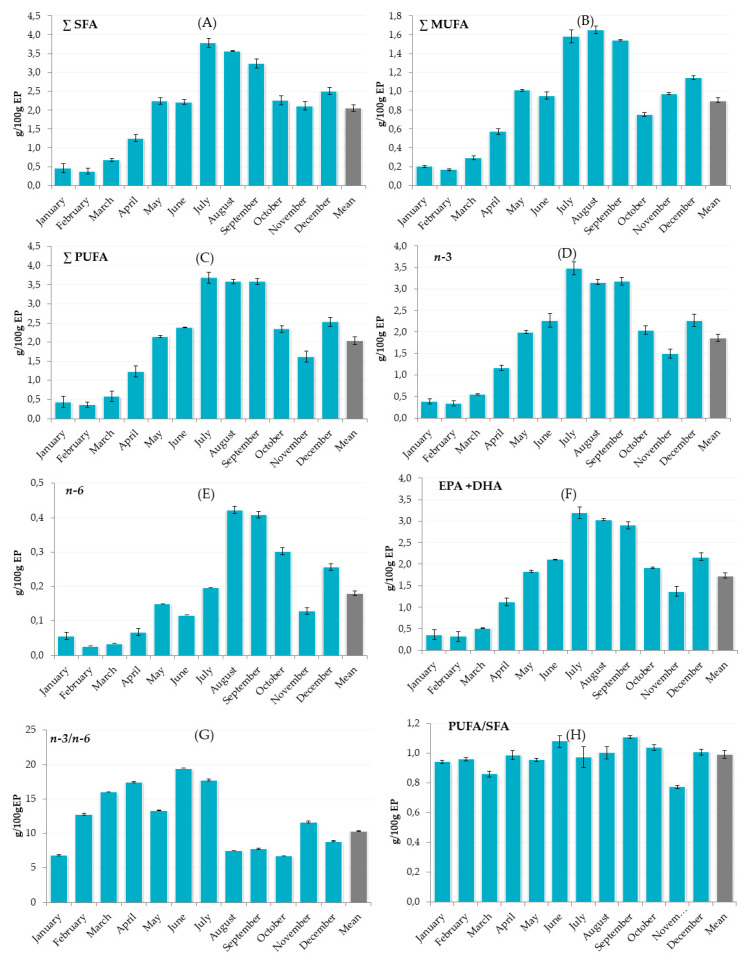
The total sum of saturated fatty acids (∑SFA, **A**), monounsaturated fatty acids (∑MUFA, **B**), polyunsaturated fatty acids (∑PUFA, **C**), total sum of *n-3* (**D**) and *n-6* (**E**), sum of eicosapentaenoic (EPA) and docosahexaenoic (DHA) fatty acids (**F**), *n*-3/*n*-6 ratio (**G**), and PUFA/SFA ratio (**H**) of sardines over 12 months.

The relative FA profile of European sardine EP (Table 3 and Figure 2) varied greatly over the year. The sum of the saturated fraction was the highest from July to September, with palmitic acid (C16:0) being predominant, followed by myristic (C14:0), and stearic fatty acid (C18:0). Palmitic acid reached levels of 2.19, 1.66, and 1.61 g/100 g of EP in July, August, and September, respectively. The lowest saturated fatty acids (SFA) were tricosylic acid (C23:0; 0.01 g/100 g) and arachidic acid (C20:0; 0.02 g/100 g).

The sum of monounsaturated FA (MUFA) and PUFA (Figure 2) were the highest during intensive feeding throughout the summer season and the lowest at the peak of the spawning period. The dominant MUFA was oleic acid (C18:1 *cis-9)*, especially during July, August, and September when 0.91, 1.02, and 0.81 g/100 g were detected, respectively. Myristoleic acid (C14:1), 10(Z)-pentadecenoic acid (C15:1), and erucic acid (C22:1 *n-9*) were not detected and the lowest MUFA was elaidic acid (C18:1 *n-9* trans). The content of PUFA was the highest, >3 g/100 g EP, during summer. In these months the dominant FA, with a share of >90% of total PUFA, were EPA (C20:5 *n-3*) and DHA (C22:6 *n-3*) and, over the year, DHA was higher than EPA. They were most prominent in July and August. During winter, particularly in January, February and March, EPA and DHA were the lowest, ranging from 0.13 to 0.31 g/100 g.

The European Food Safety Authority (EFSA) suggests the consumption of 0.25 to 0.5 g EPA and DHA in the daily diet to protect against the risk of cardiovascular disease [45]; thus, in terms of *n*-3 FA sardines can be considered a very good choice of fish all year around. Omega-6 fatty acids were found at very low levels over the year compared to omega-3, resulting in the beneficial *n-3/n-6* ratio (Figure 2G). An extremely high consumption of *n*-6 was recognized as undesirable, and a higher *n-3/n-6* ratio is considered important in the prevention of coronary heart disease, high plasma lipid levels, and cancer risks [20,46].

In terms of *n-3*/*n-6* ratio, the relative nutritional content of sardines was the most important in spring and summer seasons, being the highest in June, April and March. A higher ratio of PUFA/SFA was noticed in the summer, mostly during June and September. These results show that *S. pilchardus* is a highly valuable source of essential lipids (EPA and DHA), and is a very important part of human nutrition, as it can have benefits in terms of the suppression of cardiovascular risks (coronary heart disease and ischemic stroke), neurodegenerative diseases, and metabolic syndromes, which include visceral obesity, insulin resistance, and elevated blood pressure [41,42,43]. The adequate intake of *n*-3 and *n*-6 PUFA are recommended for human health through every stage of life, from development and maturation to aging. The appropriate intake differs based on the fact that some specific population groups might require higher levels of PUFA. In particular, these include pregnant and lactating women, infants, children, adolescents, and the elderly, whose nutrient requirements change with body composition, physical activity, or the presence of disease [44].

The findings in this research are in agreement with other studies that have investigated the effect of seasons on the FA composition of sardines, including *S. pilchardus* and *Sardinops melanostictus*. The major SFA reported in sardines were palmitic acid (C16:0), followed by myristic acid (C14:0) and stearic acid (C18:0). The dominant MUFA and PUFA were identified as palmitoleic acid (C16:1 *n*-7), oleic acid (C18:1 *n*-9), EPA, and DHA. The seasonal changes in FA result from the feeding activity of sardines and the seasonal variations in plankton, since the FA profile from sardines corresponded to that of plankton [23,24,25]. The FA profile reflects the omnivorous feeding habits of the sardines, which could be herbivory (diatom and dinoflagellate) or carnivory (copepods) depending on the availability of food [28]. In *S. pilchardus* along the Mediterranean, the highest values of PUFA, in particular, the total amount of *n-3* and *n-3/n-6* ratios over 10, were found from February to July and June to September, respectively, coinciding with the pre-spawning and feeding period [22,24,26,38,45].

## 4. Conclusions

To establish dietary recommendations and improve population health, it is essential to investigate and report on the nutritional information of food products, particularly of foods whose nutritive value changes seasonally. This study intended to investigate the nutritive components of sardines and their seasonal variation in the Central Adriatic. The lipid, fatty acid and free amino acid content of sardines (*S. pilchardus*) showed significant seasonal changes over the year. Given the affordability of sardines over the year, sardines can be considered a rich source of both AA and PUFA. In the winter season, approximately 500 mg of essential free AA can be measured in 100 g of sardine EP and, in the summer, the lipid content is the highest, >3 g of DHA and EPA. This information can be used for nutritional counseling and recommendations for individuals with specific conditions and needs. In the future, sardines may be considered a source of essential bioactive components that can benefit both nutrition-related disease treatment and physical and mental wellbeing. 

## Figures and Tables

**Table 1:** Changes in proximate composition (g/100 g EP) of sardines over 12 months (*n* = 6).

Proximate Composition	January	February	March	April	May	June	July	August	September	October	November	December	Mean
Moisture	78.6 ± 0.24	78.8 ± 0.21	78.1 ± 0.14	76.4 ± 0.21	73.8 ± 0.22	73.6 ± 0.09	69.67 ± 0.15	69.4 ± 0.24	69.8 ± 0.20	73.8 ± 0.22	74.5 ± 0.16	72.9 ± 0.09	73.0 ± 0.14
Lipid	1.40 ± 0.14	1.18 ± 0.22	1.91 ± 0.14	3.64 ± 0.84	6.23 ± 0.62	6.40 ± 0.14	10.3 ± 0.35	10.6 ± 1.14	10.1 ± 0.74	6.18 ± 0.29	5.45 ± 0.87	7.10 ± 0.80	5.87 ± 1.04
Protein	19.5 ± 0.26	20.0 ± 0.14	19.8 ± 0.24	19.0 ± 0.24	19.1 ± 0.24	19.2 ± 0.24	20.2 ± 0.24	20.1 ± 0.24	20.1 ± 0.24	19.1 ± 0.24	19.2 ± 0.24	19.5 ± 0.24	19.6 ± 0.24
Ash	1.88 ± 0.01	2.10 ± 0.01	2.30 ± 0.07	2.11 ± 0.04	1.68 ± 0.03	1.50 ± 0.03	1.45 ± 0.06	1.63 ± 0.04	1.68 ± 0.04	1.87 ± 0.07	1.91 ± 0.06	1.83 ± 0.04	1.83 ± 0.04

**Table 2:** Changes in free amino acid content in sardines over 12 months (mg/100 g wet weight) (*n* = 4).

Amino Acid	January	February	March	April	May	June	July	August	September	October	November	December	Mean
Aspartic acid	33.1 *±* 4.4	26.0 ± 2.7	40.6 ± 5.7	11.2 ± 2.9	5.67 ± 1.6	6.63 ± 2.3	5.82 ± 1.44	15.0 ± 4.1	6.19 ± 2.20	5.32 ± 2.15	6.34 ± 1.66	7.60 ± 0.10	14.1
Glutamic acid	30.3 ± 0.3	24.6 ± 0.7	38.9 ± 0.2	11.5 ± 0.9	5.30 ± 0.01	6.96 ± 0.15	7.76 ± 1.31	12.8 ± 0.2	6.07 ± 0.15	7.08 ± 0.10	7.84 ± 0.11	7.11 ± 0.11	13.9
Hydroxyproline	19.5 ± 0.3	27.8 ± 0.02	28.9 ± 0.6	13.9 ± 1.2	11.2 ± 0.1	13.1 ± 0.3	14.5 ± 0.1	15.3 ± 0.3	9.92 ± 0.22	8.12 ± 0.04	11.3 ± 0.2	9.28 ± 0.30	15.2
Serine	n.d.	n.d.	n.d.	n.d.	n.d.	n.d.	n.d.	n.d.	n.d.	n.d.	n.d.	n.d.	n.d.
Asparagine	25.0 ± 0.5	18.3 ± 0.5	32.2 ± 0.4	9.04 ± 0.92	3.92 ± 0.1	6.24 ± 0.1	7.33 ± 0.18	11.4 ± 0.4	5.17 ± 0.12	5.22 ± 0.05	7.37 ± 0.13	5.75 ± 0.10	11.4
Glycine	18.2 ± 3.1	13.9 ± 1.6	25.6 ± 2.9	8.98 ± 0.12	4.92 ± 0.2	12.0 ± 0.5	12.8 ± 0.9	16.7 ± 1.5	10.3 ± 0.6	11.1 ± 0.67	11.5 ± 1.1	12.0 ± 0.2	13.2
Glutamine	14.9 ± 2.8	10.9 ± 0.4	18.7 ± 1.8	4.40 ± 0.3	1.25 ± 0.1	1.22 ± 0.01	1.84 ± 0.07	2.84 ± 0.11	3.68 ± 0.12	1.33 ± 0.10	2.67 ± 0.02	1.84 ± 0.04	5.46
Histidine *	159 ± 2.4	149 ± 2.9	244 ± 1.9	124 ± 9.8	58.7 ± 0.6	49.5 ± 0.4	56.4 ± 0.6	60.6 ± 0.9	54.1 ± 0.2	44.2 ± 0.11	56.5 ± 0.6	66.2 ± 0.6	93.4
Threonine *	96.1 ± 0.1	60.9 ± 0.8	95.1 ± 1.0	52.5 ± 6.4	36.5 ± 1.5	79.7 ± 1.3	67.9 ± 3.2	71.0 ± 4.4	102 ± 0.2	77.9 ± 0.27	73.9 ± 0.5	58.6 ± 1.1	72.7
Alanine	13.8 ± 0.1	9.95 ± 0.3	17.6 ± 0.5	4.82 ± 0.5	2.19 ± 0.1	7.34 ± 3.01	6.25 ± 0.44	9.12 ± 0.04	6.84 ± 2.50	7.14 ± 2.98	5.35 ± 0.16	4.42 ± 0.17	7.91
Arginine	97.6 ± 0.6	76.7 ± 1.6	123 ± 0.5	47.8 ± 3.5	23.0 ± 0.4	21.8 ± 0.1	30.4 ± 0.6	36.5 ± 0.2	23.9 ± 0.1	17.9 ± 0.1	24.9 ± 0.10	21.8 ± 0.3	45.4
Proline	21.3 ± 0.02	14.5 ± 0.1	25.4 ± 0.2	8.31 ± 0.9	3.28 ± 0.4	4.49 ± 0.12	8.03 ± 0.31	10.1 ± 0.4	4.16 ± 0.02	3.87 ± 0.10	5.57 ± 0.31	3.43 ± 0.05	9.36
Tyrosine	32.7 ± 0.4	15.5 ± 0.7	32.2 ± 0.4	6.82 ± 0.4	3.09 ± 0.2	3.04 ± 0.4	4.15 ± 0.80	8.98 ± 0.19	3.09 ± 0.13	2.87 ± 0.38	5.26 ± 0.03	2.95 ± 0.01	10.0
Valine *	31.7 ± 0.7	21.1 ± 0.8	34.9 ± 0.4	10.3 ± 0.8	4.35 ± 0.5	6.26 ± 0.24	10.1 ± 0.22	14.4 ± 0.02	6.30 ± 0.18	6.11 ± 0.10	49.7 ± 0.8	6.15 ± 0.05	16.8
Methionine *	16.5 ± 1.3	10.1 ± 0.9	21.5 ± 0.2	6.38 ± 0.3	1.91 ± 0.01	4.56 ± 1.31	5.74 ± 0.04	10.6 ± 0.2	4.76 ± 0.55	4.58 ± 0.72	7.38 ± 0.23	4.90 ± 0.85	8.23
Isoleucine *	38.1 ± 0.1	31.3 ± 0.6	25.9 ± 1.1	9.79 ± 2.5	11.4 ± 0.03	6.50 ± 0.16	20.1 ± 0.01	10.8 ± 0.3	6.56 ± 0.33	7.11 ± 0.12	7.13 ± 0.15	7.48 ± 0.21	15.2
Leucine *	53.8 ± 0.2	34.8 ± 0.03	50.9 ± 0.01	16.8 ± 1.9	6.20 ± 0.02	9.12 ± 0.25	15.6 ± 0.02	20.8 ± 0.5	9.04 ± 0.06	8.55 ± 0.24	14.1 ± 0.3	9.35 ± 0.02	20.8
Phenylalanine *	33.7 ± 0.2	19.9 ± 0.2	25.4 ± 0.2	9.63 ± 1.3	3.50 ± 0.1	4.02 ± 0.11	6.16 ± 0.16	9.55 ± 0.17	4.44 ± 0.15	3.44 ± 0.11	5.64 ± 0.09	3.73 ± 0.04	10.8
Tryptophan *	25.9 ± 0.1	14.7 ± 0.2	20.7 ± 0.2	8.64 ± 0.3	5.05 ± 0.1	4.30 ± 0.12	6.48 ± 0.03	7.07 ± 0.19	4.16 ± 0.04	3.60 ± 0.03	4.41 ± 0.13	3.48 ± 0.05	9.04
Lysine *	82.9 ± 1.4	43.1 ± 1.6	53.0 ± 0.9	19.6 ± 1.4	9.83 ± 0.1	11.1 ± 0.1	26.7 ± 0.72	23.6 ± 0.3	15.5 ± 0.16	14.9 ± 0.17	15.6 ± 0.6	17.7 ± 0.1	27.8
Total	843.6	622.9	953.9	384.4	201.3	257.8	314.1	367.1	286.5	240.4	322.3	253.7	420.6

* Essential amino acids (AA); not detected (n.d.).

**Table 3:** Seasonal changes in the fatty acid composition of the sardine g/100 g wet weight (*n* = 4).

Fatty Acid (FA)	January	February	March	April	May	June	July	August	September	October	November	December	Mean
**C14:0**	0.10 ± 0.01	0.06 ± 0.01	0.11 ± 0.04	0.18 ± 0.02	0.33 ± 0.04	0.38 ± 0.04	0.54 ± 0.02	0.61 ± 0.06	0.61 ± 0.02	0.44 ± 0.03	0.29 ± 0.02	0.53 ± 0.01	0.35
**C14:1**	n.d.	n.d.	n.d.	n.d.	n.d.	n.d.	n.d.	n.d.	n.d.	n.d.	n.d.	n.d.	n.d.
**C15:0**	0.01 ± 0.01	0.01 ± 0.00	0.03 ± 0.00	0.05 ± 0.01	0.05 ± 0.01	0.09 ± 0.04	0.20 ± 0.02	0.22 ± 0.04	0.17 ± 0.03	0.10 ± 0.02	0.05 ± 0.01	0.04 ± 0.00	0.08
**C15:1**	n.d.	n.d.	n.d.	n.d.	n.d.	n.d.	n.d.	n.d.	n.d.	n.d.	n.d.	n.d.	n.d.
**C16:0**	0.21 ± 0.03	0.20 ± 0.04	0.33 ± 0.08	0.64 ± 0.12	1.12 ± 0.21	1.18 ± 0.16	2.19 ± 0.22	1.66 ± 0.19	1.61 ± 0.14	0.99 ± 0.11	1.12 ± 0.09	1.27 ± 0.14	1.04
**C16:1**	0.08 ± 0.02	0.06 ± 0.01	0.11 ± 0.01	0.14 ± 0.02	0.28 ± 0.01	0.24 ± 0.08	0.33 ± 0.04	0.39 ± 0.06	0.34 ± 0.09	0.24 ± 0.06	0.30 ± 0.02	0.38 ± 0.04	0.24
**C17:0**	0.02 ± 0.00	0.01 ± 0.00	0.01 ± 0.00	0.02 ± 0.00	0.03 ± 0.00	0.07 ± 0.01	0.12 ± 0.01	0.43 ± 0.11	0.37 ± 0.06	0.22 ± 0.04	0.10 ± 0.02	0.08 ± 0.01	0.12
**C17:1**	0.02 ± 0.00	n.d.	n.d.	0.01 ± 0.00	0.01 ± 0.00	0.01 ± 0.00	0.01 ± 0.00	0.09 ± 0.01	0.21 ± 0.08	0.10 ± 0.01	0.12 ± 0.04	0.02 ± 0.00	0.05
**C18:0**	0.11 ± 0.03	0.07 ± 0.02	0.16 ± 0.01	0.25 ± 0.03	0.53 ± 0.09	0.36 ± 0.02	0.57 ± 0.09	0.48 ± 0.03	0.30 ± 0.02	0.29 ± 0.09	0.34 ± 0.08	0.32 ± 0.07	0.31
**C18:1*n-9t***	n.d.	n.d.	n.d.	n.d.	0.01 ± 0.00	0.01 ± 0.00	0.02 ± 0.00	0.01 ± 0.00	0.01 ± 0.00	n.d.	n.d.	0.01 ± 0.00	0.01
**C18:1*n-9c***	0.08 ± 0.01	0.09 ± 0.01	0.15 ± 0.03	0.31 ± 0.02	0.58 ± 0.08	0.58 ± 0.06	0.91 ± 0.04	1.02 ± 0.08	0.81 ± 0.02	0.33 ± 0.01	0.44 ± 0.04	0.63 ± 0.03	0.49
**C18:2*n-6t***	n.d.	n.d.	n.d.	n.d.	0.01 ± 0.00	n.d.	0.02 ± 0.00	0.01 ± 0.00	0.01 ± 0.00	0.05 ± 0.01	0.01 ± 0.00	0.02 ± 0.00	0.01
**C18:2*n-6c***	0.05 ± 0.00	0.02 ± 0.00	0.03 ± 0.01	0.05 ± 0.01	0.11 ± 0.01	0.08 ± 0.01	0.14 ± 0.02	0.34 ± 0.02	0.22 ± 0.07	0.20 ± 0.03	0.09 ± 0.01	0.16 ± 0.04	0.12
**C18:3*n-6***	n.d.	n.d.	n.d.	n.d.	0.01 ± 0.00	0.01 ± 0.00	0.01 ± 0.00	0.01 ± 0.00	n.d.	0.03 ± 0.00	n.d.	0.01 ± 0.00	0.01
**C18:3*n-3***	n.d.	n.d.	n.d.	0.02 ± 0.00	0.05 ± 0.01	0.08 ± 0.01	0.10 ± 0.02	0.01 ± 0.00	0.08 ± 0.01	0.05 ± 0.01	n.d.	0.01 ± 0.00	0.03
**C20:0**	n.d.	n.d.	0.01 ± 0.00	0.02 ± 0.00	0.01 ± 0.00	0.02 ± 0.00	0.03 ± 0.01	0.03 ± 0.00	0.05 ± 0.01	0.04 ± 0.00	0.01 ± 0.00	0.04 ± 0.01	0.02
**C20:1*n-9***	0.01 ± 0.00	0.01 ± 0.00	0.02 ± 0.00	0.07 ± 0.01	0.08 ± 0.01	0.07 ± 0.01	0.11 ± 0.03	0.12 ± 0.03	0.06 ± 0.01	0.05 ± 0.01	0.07 ± 0.01	0.07 ± 0.02	0.06
**C20:2*n-6***	n.d.	n.d.	n.d.	0.01 ± 0.00	0.02 ± 0.00	0.02 ± 0.00	0.03 ± 0.00	0.03 ± 0.00	n.d.	0.03 ± 0.01	0.02 ± 0.00	0.07 ± 0.01	0.02
**C21:0**	0.01 ± 0.00	0.01 ± 0.00	0.01 ± 0.00	0.03 ± 0.01	0.04 ± 0.01	0.04 ± 0.01	0.04 ± 0.00	0.01 ± 0.00	0.05 ± 0.01	0.05 ± 0.01	0.03 ± 0.01	0.09 ± 0.01	0.03
**C20:3*n-6***	n.d.	n.d.	n.d.	n.d.	n.d.	n.d.	n.d.	n.d.	0.11 ± 0.03	n.d.	n.d.	n.d.	0.01
**C20:4*n-6***	n.d.	n.d.	n.d.	n.d.	n.d.	n.d.	n.d.	0.04 ± 0.00	0.07 ± 0.01	n.d.	n.d.	n.d.	0.01
**C20:3*n-3***	n.d.	n.d.	0.01 ± 0.00	0.01 ± 0.00	0.05 ± 0.01	0.01 ± 0.00	0.01 ± 0.00	0.02 ± 0.00	0.02 ± 0.00	0.02 ± 0.00	0.03 ± 0.00	0.02 ± 0.00	0.02
**C22:0**	0.01 ± 0.00	0.01 ± 0.00	0.02 ± 0.00	0.04 ± 0.00	0.08 ± 0.03	0.08 ± 0.01	0.03 ± 0.00	0.12 ± 0.04	0.04 ± 0.01	0.08 ± 0.01	0.05 ± 0.01	0.05 ± 0.01	0.05
**C20:5*n-3* (EPA)**	0.14 ± 0.03	0.13 ± 0.04	0.20 ± 0.04	0.50 ± 0.06	0.68 ± 0.06	0.91 ± 0.04	1.49 ± 0.16	1.16 ± 0.11	1.13 ± 0.11	0.62 ± 0.09	0.54 ± 0.11	0.85 ± 0.08	0.70
**C22:1*n-9***	n.d.	n.d.	n.d.	n.d.	n.d.	n.d.	n.d.	n.d.	0.02	n.d.	n.d.	n.d.	n.d.
**C22:2*n-6***	n.d.	n.d.	n.d.	n.d.	n.d.	n.d.	n.d.	n.d.	n.d.	n.d.	n.d.	n.d.	n.d.
**C23:0**	n.d.	n.d.	n.d.	n.d.	n.d.	n.d.	n.d.	n.d.	n.d.	0.02 ± 0.00	0.03 ± 0.01	0.02 ± 0.00	0.01
**C22:5*n-3* (DPA)**	0.02 ± 0.00	0.02 ± 0.00	0.03 ± 0.00	0.03 ± 0.00	0.06 ± 0.01	0.07 ± 0.01	0.18 ± 0.03	0.10 ± 0.01	0.17 ± 0.03	0.05 ± 0.01	0.09 ± 0.02	0.08 ± 0.02	0.07
**C24:0**	0.01 ± 0.00	0.01 ± 0.00	n.d.	0.03 ± 0.00	0.04 ± 0.00	0.01 ± 0.00	0.07 ± 0.02	0.01 ± 0.00	0.04 ± 0.01	0.04 ± 0.01	0.08 ± 0.02	0.05 ± 0.01	0.03
**C24:1*n-9***	0.01 ± 0.00	0.01 ± 0.00	0.01 ± 0.00	0.04 ± 0.01	0.05 ± 0.01	0.05 ± 0.00	0.20 ± 0.05	0.03 ± 0.01	0.08 ± 0.01	0.03 ± 0.01	0.05 ± 0.01	0.03 ± 0.01	0.05
**C22:6*n-3* (DHA)**	0.22 ± 0.03	0.19 ± 0.04	0.31 ± 0.08	0.62 ± 0.06	1.15 ± 0.11	1.20 ± 0.09	1.70 ± 0.12	1.87 ± 0.14	1.77 ± 0.11	1.30 ± 0.09	0.83 ± 0.06	1.31 ± 0.12	1.04
**Sum of FA**	1.11	0.92	1.55	3.06	5.40	5.55	9.05	8.80	8.36	5.36	4.71	6.18	5.00

Not detected (n.d.).
